# Serum uric acid level is associated with renal arteriolar hyalinosis and predicts post-donation renal function in living kidney donors

**DOI:** 10.1371/journal.pone.0320482

**Published:** 2025-03-25

**Authors:** Yuzuki Kano, Katsuyuki Tanabe, Masashi Kitagawa, Hitoshi Sugiyama, Tomoaki Yamanoi, Kasumi Yoshinaga, Kensuke Bekku, Shingo Nishimura, Motoo Araki, Jun Wada

**Affiliations:** 1 Department of Nephrology, Rheumatology, Endocrinology and Metabolism, Okayama University Graduate School of Medicine, Dentistry and Pharmaceutical Sciences, Okayama, Japan; 2 Department of Medicine, Kawasaki Medical School General Medical Center and Department of Medical Care Work, Kawasaki College of Health Professions, Okayama, Japan; 3 Department of Urology, Okayama University Graduate School of Medicine, Dentistry and Pharmaceutical Sciences, Okayama, Japan; Tribhuvan University Institute of Medicine, NEPAL

## Abstract

Major guidelines for living-donor kidney transplantation underscore the need for pre-donation evaluation of renal function, hypertension, obesity, diabetes mellitus, and albuminuria to minimize the risk of donation from marginal donors. However, validity is yet to be established. We retrospectively investigated the relationship between clinical characteristics and histological indices in baseline renal biopsies (0-h biopsies) and whether these parameters could predict renal function in living kidney donors one year post-donation. Seventy-six living kidney donors were recruited for this study. In histological analyses, glomerulosclerosis, arteriosclerosis, arteriolosclerosis, arteriolar hyalinosis, and interstitial fibrosis and tubular atrophy scores/indices were evaluated. Post-donation serum creatinine levels in kidney donors with arteriolar hyalinosis were significantly higher than those in individuals without arteriolar hyalinosis. There was a significant correlation between baseline serum uric acid levels and the arteriolar hyalinosis index, with baseline uric acid level identified as an independent factor for hyalinosis in multiple regression analysis. Additionally, the serum uric acid level was a significant prognostic factor for post-donation serum creatinine after adjustment for baseline clinical parameters. These data demonstrate that pre-donation serum uric acid levels are associated with arteriolar hyalinosis in the kidney and could predict a decline in renal function during the first year after donation in living kidney donors.

## Introduction

Chronic kidney disease (CKD) is a common and increasing global public health concern. Kidney transplantation remains the mainstay of renal replacement therapy for end-stage kidney disease (ESKD), because it can achieve longer survival and superior quality of life compared with dialysis therapy [1]. However, owing to the shortage of organs for kidney transplantation, the transplant community continues to seek ways to increase organ donation from living donors. Such challenging issues have led to the expansion of donation to so-called “marginal donors” whose kidneys would have been previously considered unsuitable. Because considerable attention must be paid to the potential risk of ESKD in living kidney donors, the Amsterdam Forum on the Care of the Live Kidney Donor proposed a recommendation for kidney donation from marginal donors [2]. Age, blood pressure, body mass index (BMI), glomerular filtration rate (GFR), proteinuria, and glucose tolerance are included in the donation criteria. Current guidelines for kidney donation from marginal donors worldwide generally follow the Amsterdam criteria, with partial modification in some areas. For example, the guidelines of the Japanese Society for Clinical Renal Transplantation allow kidney donation from well-controlled diabetic donors (hemoglobin A1c [HbA1c] < 6.5%) without insulin therapy and albuminuria [3]. According to these guidelines, kidney transplantation using selected marginal living donors has achieved acceptable outcomes [4–6]. The validity of such donation criteria from marginal living donors should be confirmed in prospective long-term studies, and it is important to identify and manage risk factors that are potentially harmful to the kidney, but are not included in the donation criteria from marginal donors.

Uric acid (UA) is the final product of purine metabolism in humans, and elevated serum UA levels are classically associated with gout. With an ongoing global pandemic of metabolic syndrome, the prevalence of hyperuricemia is increasing in the Asian population [7,8]. Hyperuricemia has recently been found to be a risk factor for major cardiovascular diseases, including myocardial infarction and stroke, concurrent with hypertension, dyslipidemia, and diabetes mellitus. This association between hyperuricemia and cardiovascular disease is probably due to UA-induced endothelial dysfunction [9]. Recently, hyperuricemia has been reported to be related to reduced nitric oxide production [10], endoplasmic reticulum stress [11], endothelial-to-mesenchymal transition [12], and enhanced procoagulant activity in endothelial cells [13]. Furthermore, previous studies have reported that hyperuricemia is independently associated with CKD progression [14,15]. According to renal biopsy studies, increased serum UA level can exacerbate renal arteriolopathy in patients with CKD [16,17]. Moreover, recent evidence has recognized hyperuricemia as a trigger for abnormal immune responses in the kidney, which can cause vascular and tubulointerstitial inflammation, leading to CKD progression [18]. Since urinary urate excretion is reduced after kidney donation due to a partially reduced GFR, hyperuricemia has been a concern in living kidney donors. Indeed, a large cohort study demonstrated that kidney donation is associated with a modest increase in the incidence of gout [19]. Although hyperuricemia is not included in the Amsterdam criteria, 2017 Kidney Disease Improving Global Outcomes Clinical Practice Guideline on the Evaluation and Care of Living Kidney Donors refers to the effect of serum UA levels on the risk of gout in living kidney donors [20]. However, the effects of pre-donation hyperuricemia on renal histology and the prognosis of living donors have not yet been fully determined.

In this study, we identified serum UA level as a factor associated with renal arteriolar hyalinosis in baseline (0-h) kidney biopsies to evaluate the status of donor kidneys and found that UA levels predicted renal function at one year after donation in living kidney donors. This study highlights the importance of managing serum UA levels before kidney donation.

## Materials and methods

### Participants

Data were collected from consecutive living kidney donors who underwent nephrectomy at Okayama University Hospital between May 2009 and May 2017. An opt-out notice regarding this retrospective study was provided, and donors who chose to opt out were excluded from the data collection. Finally, data from 76 kidney donors were analyzed. Among the participants, all living kidney transplantations were performed between recipients and their relatives. All the participants underwent a thorough medical evaluation prior to donation as a prescheduled series of tests, and the donation strictly followed guidelines of the Japanese Society for Clinical Renal Transplantation; that is, all participants were aged < 80 years and had a BMI of < 32 kg/m^2^, creatinine clearance (CCr) of > 70 mL/min per 1.73m^2^ of body surface area from 24-h urine collection, urinary albumin excretion of < 30 mg/day, well-controlled blood pressure (<140/90 mmHg without medication or < 130/80 mmHg with medication), and HbA1c levels of < 6.5% (National Glycohemoglobin Standardization Program) with or without oral medication. 0-h biopsy specimens and laboratory data were obtained from all participants. All procedures in the present study were performed in accordance with the institutional and national ethical guidelines for human studies, and guidelines proposed in the Declaration of Helsinki. The ethics committees of Okayama University Graduate School of Medicine, Dentistry and Pharmaceutical Sciences and Okayama University Hospital approved the study (approval number: 1709-043).

### Laboratory measurement

Pre-donation demographic data were obtained from medical records. Age, sex, smoking status, BMI, systolic blood pressure (SBP), diastolic blood pressure (DBP), and medications were used as baseline clinical data at donor registration. Laboratory data immediately pre-donation, including serum creatinine (Cr), estimated GFR (eGFR), CCr, blood urea nitrogen, serum UA, HbA1c, total cholesterol, and urinary protein excretion were obtained from the medical records as baseline laboratory parameters. The serum Cr level at the visit one year post-donation were measured to estimate post-donation renal function. Serum Cr, blood urea nitrogen, total cholesterol, and urinary protein levels were measured using a clinical biochemistry analyzer, JCA-BM8040 (JEOL Ltd., Tokyo, Japan), whereas the HbA1c level was measured using an automatic glycohemoglobin analyzer, HLC-723G11 (Tosoh Bioscience LLC, Tokyo, Japan) at Okayama University Hospital. Mean arterial pressure (MAP) was calculated as follows: MAP =  DBP +  (SBP −  DBP)/3. The eGFR was calculated according to the simplified version of the Modification of Diet in Renal Disease formula for Japanese [21]: $\text{eGFR = 194 ×}{\left(\text{sCr}\right)}^{\text{-1}.094}\text{× }{\left(\text{age}\right)}^{\text{-0}.287}$ (if female, ×  0.739). Serum Cr levels during the visit one year after donation were used as post-donation Cr. Changes in serum Cr levels (mg/dL) was calculated using Cr levels at baseline (pre-donation) and one year after donation (post-donation) as follows: ΔCr (mg/dL) =  [post-donation Cr] −  [pre-donation Cr]. We accessed the medical records to collect data for each participant from Jan. 8, 2018 to Mar. 27, 2020.

### Histopathological examinations

Needle-biopsied kidney samples were obtained from the outer cortex of the donated kidney at the time of donation (0-h biopsy). Kidney samples were stained with periodic acid–Schiff, periodic acid silver methenamine, and Masson’s trichrome, and were examined using light microscopy. In a histological analysis of the specimens, glomerulosclerosis (GS), arteriosclerosis (AS), arteriolosclerosis (As), arteriolar hyalinosis (AH), and interstitial fibrosis and tubular atrophy (IFTA) indices were semi-quantified using a scoring system (0–3) in accordance with previous reports [22–24]. GS was expressed as the percentage of globally sclerotic glomeruli among the total number of glomeruli. Arteriosclerosis was calculated as the ratio of intimal thickness to media thickness on both sides of the interlobular or arcuate artery and scored as follows: score 0 =  no intimal thickening, score 1 =  intima/media ratio <  1, and score 2 =  intima/media ratio ≥  1. The arteriolosclerosis index was calculated using the following formula: $\text{As index = arteriolar lumen diameter/}\left(\text{outer diameter - lumen diameter}\right)\text{/2}$. Arteriolar hyalinosis was scored by the percentage of hyaline thickness of the whole arteriolar wall, as follows: score 0 =  no hyalinosis, score 1 =  partial, score 2 =  < 50%, and score 3 =  ≥ 50% of the whole arteriolar wall. Additionally, the AH index was calculated using the following formula: $\text{AH index = }\left(\text{n0 × 0 + n1 × 1 + n2 × 2 + n3 × 3}\right)\text{/N}$. Here, n1, n2, and n3 indicate the number of arterioles with hyalinosis scores of 1–3, respectively, and N indicates the total number of arterioles. Interstitial fibrosis and tubular atrophy were assessed using the percentage of fibrotic area and scored as follows: score 0, absent; score 1, < 25%; score 2, 25%–50%; and score 3, > 50% of the total cortical area.

### Statistical analysis

All statistical analyses were performed using JMP Pro 17 software (SAS Institute, Cary, NC, USA). Continuous variables are expressed as mean ±  standard deviation, and categorical variables are expressed as numbers with percentages (%). Statistical significance was defined as a two-sided p-value of < 0.05. Differences between the two groups were tested using the *t*-test for parametric data and the Mann–Whitney U test for non-parametric data. Adjusted odds ratio of the presence of AH for a 1 mg/dL increase in serum UA was calculated using multiple logistic regression analysis, after adjusting for the potential confounding factors. The Spearman’s rank correlations were used to determine the relationship between the clinical parameters and histological indices. Multiple regression analysis was performed to evaluate the contributions of serum UA to AH index and post-donation renal function, after adjusting for potential confounding factors.

## Results

### Characteristics of participants and histological scores/indices

Table 1 summarizes the characteristics of 76 participants included in this study. The mean age was 59 ±  9 years, with 45% of the participants being male. The BMI was 23.2 ±  2.7 kg/m^2^ and no participant had a BMI of > 30 kg/m^2^. In accordance with the inclusion criteria, all participants had a CCr of > 70 mL/min. The mean CCr was 93 ±  19 mL/min, with 17% of the participants having CCr of 70–80 mL/min. Well-controlled hypertension with medication was observed in 22.4% (17/76) of the participants, and renin-angiotensin system inhibitors were used in 18.4% (14/76). Well-controlled diabetes mellitus with oral medication was observed in 9.2% (7/76) of the participants. The serum UA level was 5.0 ±  1.3 mg/dL. Hyperuricemia, defined as a serum UA level of > 7 mg/dL, was observed in 11.8% (9/76) of the participants. Only two participants (2.6%) took UA lowering drugs at baseline. Serum Cr one year after donation were obtained from 72 participants. The changes in serum Cr (ΔCr) between pre-donation and one year post-donation was calculated to be 0.36 ±  0.12 mg/dL.

**Table 1:** Clinical and laboratory characteristics of study participants.

Baseline characteristics (n = 76)	Mean ± SD or n (%)
Age (y)	58 ± 9
Male, n (%)	34 (44.7)
Body mass index (kg/m^2^)	23.2 ± 2.7
Current smoking (%)	19 (25.0)
Systolic blood pressure (mmHg)	124 ± 16
Diastolic blood pressure (mmHg)	77 ± 11
Mean arterial blood pressure (mmHg)	93 ± 11
Anti-hypertensive drugs (%)	17 (22.4)
Serum creatinine (mg/dL)	0.72 ± 0.15
eGFR (mL/min/1.73m^2^)	77 ± 13
Creatinine clearance (mL/min)	93 ± 19
Blood urea nitrogen (mg/d)	13.6 ± 3.3
Serum albumin (g/dL)	4.5 ± 0.3
Serum uric acid (mg/dL)	5.0 ± 1.3
Uric acid-lowering drugs (%)	2 (2.6)
Total cholesterol (mg/dL)	214.8 ± 35.7
Hemoglobin A1c (%)	5.7 ± 0.5
Anti-diabetic drugs (%)	7 (9.2)
Urinary protein excretion (g/day)	0.06 ± 0.04
**Renal function one year after donation (n = 72)**	**Mean ± SD**
Serum creatinine (mg/dL)	1.08 ± 0.23
eGFR (mL/min/1.73m^2^)	49 ± 8

eGFR, estimated glomerular filtration rate; SD, standard deviation.

**Fig 1:**
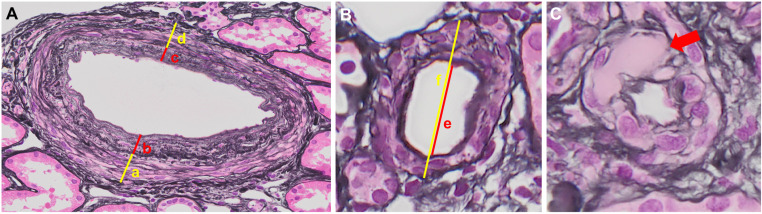
Representative images for artery and arterioles for 0-h kidney biopsy specimens (periodic acid silver methenamine-stained). A) Arteriosclerosis (AS) was scored as 0: no intimal thickening, 1: (b +  c)/(a + d) <  50%, 2: (b +  c)/(a +  d) >  50%. B) Arteriolosclerosis (As) index was calculated as e/(f −  e)/2. C) Arteriolar hyalinosis (AH) was scored by the percentage of hyaline thickness (red arrow) in arteriolar wall as 0: none; 1: partial; 2: < 50%; and 3: > 50%. See the “Methods” section for calculation of indices.

Representative images of arterial and arteriolar changes in 0-h kidney biopsy specimens are presented in Fig 1, and histological scores/indices based on the biopsy findings are listed in Table 2. GS, AS score, As index, AH index, and IFTA scores were evaluated in 69, 66, 50, 66, and 69 of the 76 participants, respectively. The AS and IFTA scores were predominantly 1–2, whereas the AH score showed a bimodal pattern, with peaks at 0 and 3.

**Table 2:** Histological findings at 0-h kidney biopsy.

Histological findings	Mean ± SD or n (%)
Glomerulosclerosis [GS] (%) (n = 69)	13.5 ± 14.3
Arteriosclerosis [AS] score (n = 50)	
0	6 (12.0)
1	11 (22.0)
2	33 (66.0)
average	1.24 ± 0.70
Arteriolosclerosis [As] index* (n = 66)	2.66 ± 0.68
Arteriolar hyalinosis [AH] score (n = 66)	
0	45 (68.2)
1	1 (1.5)
2	2 (3.0)
3	18 (27.3)
Index**	0.17 ± 0.33
Interstitial fibrosis and tubular atrophy [IFTA] score (n = 69)	
0	4 (5.8)
1	46 (66.6)
2	17 (24.6)
3	2 (2.9)
average	1.25 ± 0.60

AS, AH, and IFTA lesions were scored 0–3. * As index was calculated based on outer and lumen diameters of arterioles. **AH index was calculated based on the number of arterioles with AH scores of 1–3. See the “Methods” section for details. SD, standard deviation.

### Effect of histological findings on post-donation renal function

**Fig 2:**
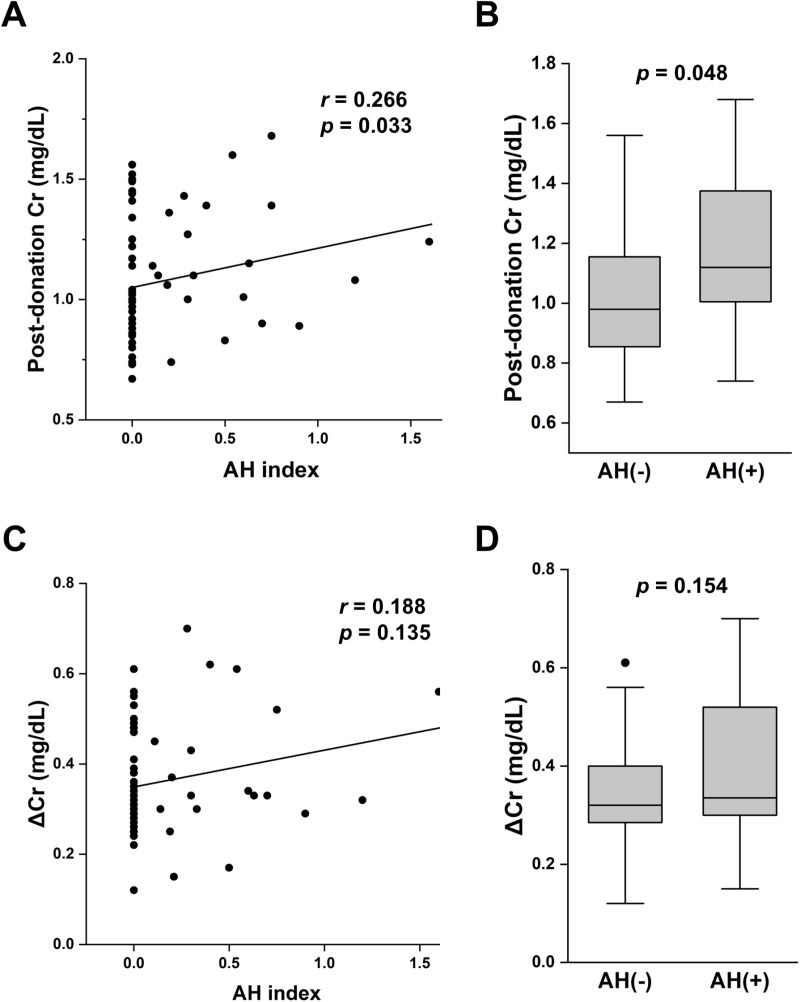
Relationship between arteriolar hyalinosis and post-donation renal function. A) Scatter plot shows significant correlation between post-donation serum creatinine and AH index. B) Serum creatinine levels one year after donation in participants with or without AH (AH score > 0). C) Scatter plot shows insignificant trend in relationship between changes in serum creatinine levels (ΔCr) between pre-donation and post-donation and AH index. D) ΔCr between pre-donation and post-donation in participants with or without AH. Cr, creatinine; AH, arteriolar hyalinosis evaluated in 0-h biopsy specimens.

First, we investigated the histological scores/indices at 0-h kidney biopsy that could affect renal function one year after kidney donation. Among the histological scores/indices, only the AH index showed significant positive association with post-donation serum Cr (*r* =  0.266, *p* =  0.033) (Table 3 and Fig 2A). Furthermore, serum Cr levels at one year post-donation were significantly higher in participants with AH lesions (AH scores >  0) than those in participants without AH lesions (*p* =  0.048) (Fig 2B), although there was no significant difference in baseline serum Cr between participants with and without AH lesions (0.77 ±  0.15 and 0.69 ±  0.16 mg/dL, *p* =  0.062). In contrast, there was a positive relationship between ΔCr and AH index, but the relationship was not statistically significant (Table 3 and Fig 2C). Changes in serum Cr levels tended to be higher in participants with AH lesions, but the difference was not significant (Fig 2D). The GS, As index, and IFTA scores did not correlate with post-donation renal function. Although the average AS was negatively associated with post-donation serum Cr, it did not correlate with ΔCr (Table 3). These results suggest that AH lesions in the kidneys at the time of donation could affect the degree of post-donation decline in renal function in living kidney donors.

**Table 3:** Correlations between histological score/indices at 0-h biopsy and post-donation renal function.

	Post-donation Cr		ΔCr	
	*r*	*P*	*r*	*P*
AH index	0.266	0.033*	0.188	0.135
GS (%)	0.088	0.477	0.075	0.548
AS average	−0.340	0.018*	−0.099	0.499
As index	−0.059	0.646	−0.072	0.572
IFTA score	0.060	0.632	0.008	0.949

Cr, serum creatinine (mg/dL); ΔCr, changes in serum Cr during the first year after donation (mg/dL); AH, arteriolar hyalinosis; GS, glomerulosclerosis; AS, arteriosclerosis; As, arteriolosclerosis; IFTA, interstitial fibrosis and tubular atrophy. * Indicates statistical significance.

### Relationship between serum UA levels and histological scores/indices

**Fig 3:**
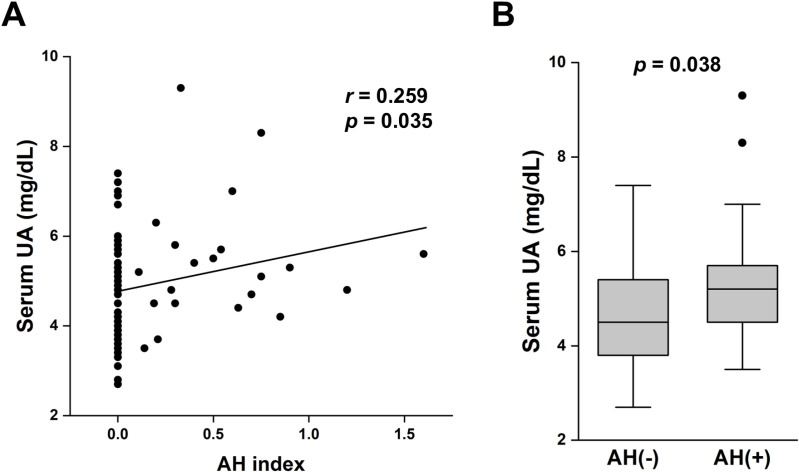
Relationship between serum uric acid (UA) and arteriolar hyalinosis. A) Scatter plot showing significant correlation between serum UA and AH index. B) Serum UA levels in participants with or without AH (AH index > 0). AH, arteriolar hyalinosis evaluated in 0-h biopsy specimens.

Next, we investigated the baseline clinical and laboratory parameters that could affect the histologic scores/indices at 0-h kidney biopsy. Although MAP, eGFR, HbA1c, and total cholesterol were not associated with any histological scores/indices (Table 4), serum UA level significantly correlated with the AH index (*r* =  0.259, *p* =  0.035) (Fig 3A). In multiple regression analysis, pre-donation serum UA was an independent predictor of AH index after adjusting for age, eGFR, smoking, and MAP (Table 5). Furthermore, serum UA levels were significantly higher in an AH (+) group with AH score > 0 compared to those in an AH (−) group (*p* =  0.038) (Fig 3B). The odds ratio of AH lesions for a 1 mg/dL increase in serum UA was 1.65 after adjusting for age (*p* =  0.028) and 1.57 after adjusting for age, eGFR, smoking, and MAP (*p* =  0.053) (Table 5). There was a negative correlation between serum UA and average AS (*r* =  − 0.331, *p* =  0.018), but there was no significant difference in UA levels between the AS (+) with an AS score > 0 and AS (−) groups (not shown). There were no significant correlations between serum UA levels and the GS and IFTA scores.

**Table 4:** Correlations between clinical parameters at baseline and histological score/indices at 0-h biopsy.

	AH index		GS (%)		Average AS		As index		IFTA score	
	*r*	*P*	*r*	*P*	*r*	*P*	*r*	*P*	*r*	*P*
MAP	−0.089	0.474	−0.218	0.071	0.181	0.207	0.078	0.533	0.143	0.238
eGFR	−0.123	0.323	−0.154	0.205	0.143	0.32	0.079	0.524	−0.194	0.109
UA	0.259	0.035*	0.051	0.673	−0.331	0.018*	−0.092	0.462	0.089	0.466
HbA1c	0.138	0.272	−0.013	0.913	0.09	0.536	0.108	0.388	0.198	0.104
T-Cho	0.036	0.776	−0.097	0.429	0.273	0.055	−0.118	0.347	0.070	0.570

AH, arteriolar hyalinosis; GS, glomerulosclerosis; AS, arteriosclerosis; As, arteriolosclerosis; IFTA, interstitial fibrosis and tubular atrophy; MAP, mean arterial pressure (mmHg); eGFR, estimated glomerular filtration rate (mL/min/1.73m^2^); UA, serum uric acid (mg/dL); HbA1c, hemoglobin A1c (%); T-Cho, total cholesterol (mg/dL). * Indicates statistical significance.

**Table 5:** Multiple regression for serum uric acid (UA) and arteriolar hyalinosis (AH) index (left) and odds ratio of AH (score > 0) for 1 mg/dL increase in serum UA (right).

	AH index		AH (+)	
	β (95% CI)	*P*	Odds ratio	*P*
Age-adjusted	0.259 (0.188 – 0.331)	0.034*	1.65 (1.05 – 2.58)	0.028*
Multiple-adjusted	0.263 (0.201 – 0.327)	0.041*	1.57 (0.99 – 2.48)	0.053

In multiple-adjusted model, data were adjusted for age, estimated glomerular filtration rate, current smoking, and mean arterial pressure. CI, confidence interval. * Indicates statistical significance.

### Relationship between serum UA levels and post-donation kidney function

**Fig 4:**
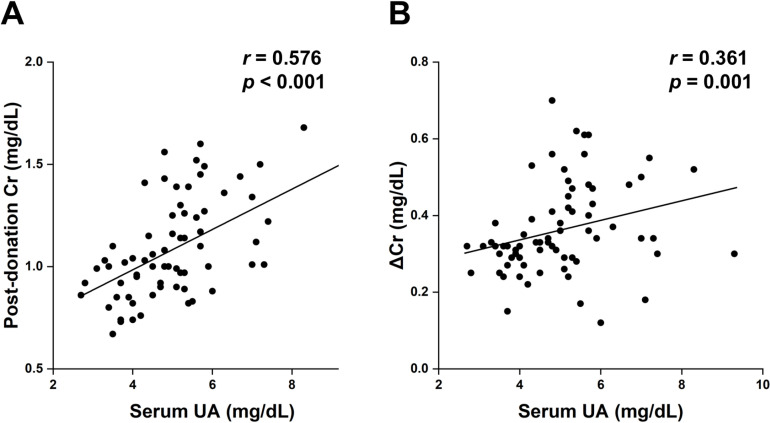
Relationship between serum uric acid (UA) and post-donation renal function. A) Significant correlation between pre-donation serum UA and post-donation (one year after donation) serum creatinine (Cr) levels. B) Significant correlation between pre-donation UA and changes in serum creatinine (ΔCr) levels between pre-donation and post-donation.

Finally, we examined the effect of pre-donation serum UA levels on post-donation renal function. Among some clinical and laboratory parameters, serum UA and eGFR at baseline were significantly correlated with post-donation serum Cr and ΔCr during the first year post-donation (Table 6 and Fig 4). Based on a multiple regression analysis, serum UA levels at baseline were considered a significant prognostic factor for greater post-donation serum Cr and ΔCr after adjusting for the potential confounding factors, including age, eGFR, smoking, MAP, and urinary protein excretion (Table 7). These results suggest that pre-donation serum UA levels have an important effect on renal function after kidney donation, independent of pre-donation renal function in living kidney donors.

**Table 6:** Correlations between clinical parameters at baseline and post-donation renal function.

	Post-donation Cr		ΔCr	
	*r*	*P*	*r*	*P*
MAP	−0.176	0.139	−0.099	0.406
eGFR	−0.594	<0.001*	−0.254	0.031*
UA	0.576	<0.001*	0.361	0.001*
HbA1c	−0.080	0.508	0.042	0.729
T-Cho	−0.192	0.106	−0.127	0.287

Cr, serum creatinine (mg/dL); ΔCr, changes in serum Cr during the first year after donation (mg/dL); MAP, mean arterial pressure (mmHg); eGFR, estimated glomerular filtration rate (mL/min/1.73m^2^); UA, serum uric acid (mg/dL); HbA1c, hemoglobin A1c (%); T-Cho, total cholesterol (mg/dL). * Indicates statistical significance.

**Table 7:** Multiple regression analysis for serum uric acid levels at baseline and post-donation renal function.

	Post-donation Cr		ΔCr	
	β (95% CI)	*P*	β (95% CI)	*P*
Model 1	0.541 (0.505 – 0.578)	<0.001*	0.293 (0.234 – 0.314)	0.011*
Model 2	0.414 (0.383 – 0.445)	<0.001*	0.249 (0.228 – 0.269)	0.033*
Model 3	0.417 (0.386 – 0.449)	<0.001*	0.268 (0.246 – 0.289)	0.031*

Model 1: adjusted for age, Model 2: adjusted for age and estimated glomerular filtration rate (eGFR), Model 3: adjusted for age, eGFR, current smoking, mean arterial pressure, and urinary protein excretion. Cr, serum creatinine (mg/dL); ΔCr, changes in serum Cr during the first year after donation (mg/dL); CI, confidence interval. * Indicates statistical significance.

## Discussion

In the present study, we focused on the effects of pre-donation serum UA levels on histological findings in 0-h kidney biopsy specimens and post-donation kidney function one year after donation.

Since kidney donors experience a reduction in renal function after uninephrectomy, the risk factors for CKD, including high blood pressure, obesity, and hyperglycemia, need to be strictly managed, especially in younger donors. Hyperuricemia is a potentially modifiable risk factor for the development and progression of CKD [25]. With the increased prevalence of obesity and metabolic syndrome, hyperuricemia has become an increasingly important factor for kidney donors [26]. According to the National Health and Nutrition Examination Survey 2007–2008, hyperuricemia increases the risk of ESKD by 4.5 and 9 times in men and women, respectively [27]. However, the role of hyperuricemia as a therapeutic target in CKD remains controversial. In particular, there is little evidence that pre-existing hyperuricemia affects the course of CKD in kidney donors, although new-onset hyperuricemia after donation is a known risk factor for the development of gout [19]. Considering the evidence from basic and clinical studies that hyperuricemia can induce endothelial dysfunction in cardiovascular diseases [9], the contributions of hyperuricemia to renal dysfunction and underlying mechanisms need to be specifically examined in kidney donors to prevent CKD progression.

**Fig 5:**
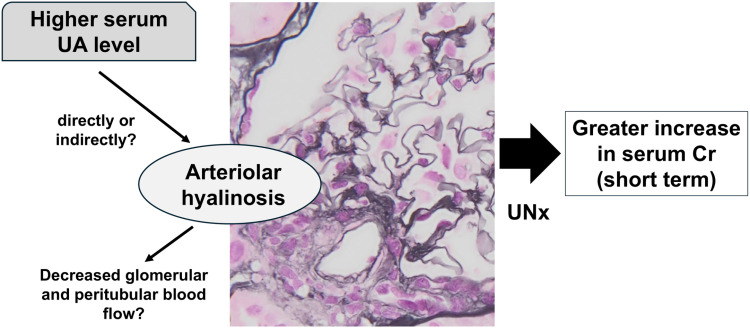
Hypothetical relationship between serum uric acid, arteriolar hyalinosis, and post-donation renal function. UA, uric acid; Cr, creatinine; UNx, unilateral nephrectomy.

Notably, this study included only 11.8% of participants with serum UA > 7 mg/dL, and a majority of the participants had serum UA levels within normal range. Nevertheless, this study suggested an association between serum UA levels and AH index in 0-h biopsy specimens in kidney donors, consistent with findings from a previous report on patients with CKD [17]. Furthermore, a significant association between the AH index and post-donation serum creatinine levels was observed. The clinical significance of AH in patients with CKD has been well demonstrated. For example, a higher AH index predicts an increase in albuminuria and decline in GFR in patients with type 2 diabetes mellitus [28]. Based on these results from the present study, pre-existing higher serum UA levels can be presumed to have adverse effects on renal function after kidney donation due to renal arteriolar damage. This hypothesis can also be explained by previous evidence showing that higher serum UA levels are significantly correlated with higher vascular resistance in the renal afferent arteriole [29]. A similar association among higher UA levels, AH, and renal prognosis was reported in a recent study involving patients with immunoglobulin A nephropathy [16]. However, the mechanisms by which hyperuricemia induces AH are not fully understood. The proposed mechanisms of hyperuricemia-induced vascular injury include endothelial dysfunction and vascular smooth muscle cell proliferation via increased intracellular oxidative stress [9]. Increased serum UA levels have been shown to cause renal afferent arteriolar thickening in a rat model [30]. In addition to its effects on donor renal function, two recent studies from the Netherlands and Japan have investigated the effects of AH in pre-transplant biopsies on graft survival in kidney recipients. None of the studies showed significant statistical correlations between AH and long-term graft survival [31,32]. However, the study among Japanese patients demonstrated significantly lower long-term renal function in living donors who had kidneys with AH [32]. Therefore, AH in 0-h biopsy specimen may have a greater effect on renal function in kidney donors than on graft function in kidney recipients. Fig 5 summarizes the hypothetical relationship between higher serum UA, AH, and post-donation renal function. Although the correlation between AH index and post-donation serum Cr was shown, significant relationship between AH index and ΔCr was not observed. Therefore, higher serum UA might affect post-donation renal function independent of AH.

Unfortunately, it is generally difficult to evaluate AH before kidney donation unless renal biopsy is routinely performed in potential kidney donors. Based on the significant association between AH in 0-h biopsy specimens and higher serum UA levels before donation in the present study, it would be plausible to consider potential kidney donors with hyperuricemia at risk of having renal AH. As this study also indicated a higher risk of renal function decline in kidney donors with higher pre-donation UA levels, serum UA may need to be appropriately managed before kidney donation, and target UA levels may be considerably lower than 7 mg/dL, which is diagnosed as hyperuricemia. A recent report demonstrated that higher baseline UA levels were associated with not only a lower eGFR but also a higher incidence of adverse events including cardiovascular disease for 60 months [33]. However, whether pharmacological intervention for hyperuricemia before kidney donation is effective in preventing the progression of renal AH and post-operative decline in renal function remains unknown. Among UA-lowering drugs, allopurinol has been suggested to improve endothelial function [34], but its protective effects on renal function have been questioned [35]. Recently, empagliflozin, a sodium-glucose co-transporter 2 (SGLT2) inhibitor that prevents decline of eGFR [36], was reported to have a UA-lowering effect in patients with CKD [37]. To date, the efficacy and safety of SGLT2 inhibitors in kidney donors have not been reported. Therefore, the best way to reduce the post-donation risk of renal dysfunction associated with hyperuricemia-induced renal AH may be strict management of serum UA prior to kidney donation through both lifestyle and pharmacological interventions, although the target UA level remains unclear.

In simple regression analysis, significant negative association between serum UA and average AS was also observed. This result might be caused by the fact that female participants, who generally have lower serum UA levels, had higher AS scores in this study (*p* =  0.043) due to unknown reasons. Therefore, such negative association is probably not be generalized in population with higher AS scores in male.

This study has some limitations. First, the study design was retrospective; thus, it could not demonstrate causal associations between serum UA, AH, and post-donation renal function. Although multiple regression analyses adjusted for baseline blood pressure and eGFR were performed to confirm these associations, there might be undetermined confounding factors. Second, the sample size was relatively small. The results obtained in this study should be verified in larger cohorts. Additionally, the observation period for renal function was only 1 year. A substantially longer evaluation period is needed to clarify whether hyperuricemia has a significant effect on the risk of ESKD in living kidney donors. Finally, the baseline serum UA levels were determined at one time point. Thus, long-term variations in the UA levels before kidney donation were not reflected in this study. Nevertheless, this study emphasizes the importance of pre-donation UA levels in the management of living kidney donors.

In conclusion, hyperuricemia is associated with AH and is a potential risk factor for one year post-donation renal dysfunction in living kidney donors. Furthermore, based on clinicopathological associations, higher serum UA levels should be included in the evaluation of candidate kidney donors as alternative markers for AH in the kidneys.

## Supporting information

S1 Appendix Human participants research checklist. (DOCX)
